# Socioeconomic inequalities in the food environment and body composition among school-aged children: a fixed-effects analysis

**DOI:** 10.1038/s41366-021-00934-y

**Published:** 2021-08-13

**Authors:** Famke J. M. Mölenberg, Joreintje D. Mackenbach, Maartje P. Poelman, Susana Santos, Alex Burdorf, Frank J. van Lenthe

**Affiliations:** 1 Department of Public Health, Erasmus MC, University Medical Centre Rotterdam, Rotterdam, The Netherlands; 2 The Generation R Study Group, Erasmus MC, University Medical Centre Rotterdam, Rotterdam, The Netherlands; 3 Department of Epidemiology and Data Science, Amsterdam Public Health Research Institute, Amsterdam UMC, Vrije Universiteit Amsterdam, Amsterdam, The Netherlands; 4 Chair Group Consumption and Healthy Lifestyles, Wageningen University & Research, Wageningen, The Netherlands; 5 Department of Paediatrics, Erasmus MC, University Medical Centre Rotterdam, Rotterdam, The Netherlands; 6 Faculty of Geosciences, Department of Human Geography and Spatial Planning, Utrecht University, Utrecht, The Netherlands

**Keywords:** Risk factors, Epidemiology, Nutrition, Preventive medicine

## Abstract

**Background:**

There is limited evidence regarding socioeconomic inequalities of exposure to the food environment and its contribution to childhood obesity.

**Methods:**

We used data from 4235 children from the Generation R Study, a large birth-cohort conducted in the city of Rotterdam, The Netherlands. We included 11,277 person-observations of body mass index (BMI) and 6240 person-observations of DXA-derived fat mass index (FMI) and fat-free mass index (FFMI) when children were between 4 and 14 years. We applied linear regression models to evaluate changes in the relative and absolute exposure of fast-food outlets, and the healthiness of the food environment within 400 m from home by maternal education. Furthermore, we used individual-level fixed-effects models to study changes in the food environment to changes in BMI, FMI and FFMI.

**Results:**

Children from lower educated mothers were exposed to more fast-food outlets at any time-point between the age of 4 and 14 years. Over a median period of 7.1 years, the absolute (0.6 fast-food outlet (95% CI: 0.4–0.8)) and relative (2.0%-point (95% CI: 0.7–3.4)) amount of fast-food outlets increased more for children from lower as compared to higher educated mothers. The food environment became more unhealthy over time, but no differences in trends were seen by maternal education level. Changes in the food environment were not associated with subsequent changes in BMI, FMI and FFMI. For children from lower educated mothers not exposed to fast-food at first, we found some evidence that the introduction of fast-food was associated with small increases in BMI.

**Conclusions:**

Our findings provide evidence of widening inequalities in exposure to fast-food in an already poor food environment. Access to more fast-food outlets does not seem to have an additional impact on BMI in contemporary contexts with ubiquitous fast-food outlets.

## Introduction

Childhood obesity is a major public health concern due to its widespread prevalence and rapid increase in the past decades [1, 2]. The food environment has also changed considerably, mainly towards a higher exposure of food outlets in residential areas [3], and a higher offer of high-energy and ultra-processed foods [4, 5]. It is likely that food environments have contributed to rising childhood obesity. However, evidence for a causal influence of changing food environments on childhood obesity is limited [6–9]. Studies have been mainly cross-sectional, and cannot rule out the effects of residential self-selection based on preferences and resources related to both the food environment and obesity. Some studies have offset some of the selection processes by applying fixed-effects models with repeated measurements taking into account measured time-varying variables (e.g., income) and unmeasured time-invariant variables (e.g., neighbourhood preference) [10–14]. We identified one study in children, and this US study counterintuitively showed that the increase in fast-food outlets around home was associated with small reductions in body mass index (BMI) [10]. This finding warrants further exploration in other settings.

Changes in the food environment and their impact on obesity have been hypothesised to differ between individuals from higher and lower socioeconomic groups. A systematic review of 21 studies showed that fast-food access was higher in more-deprived compared to less-deprived areas [15]. Individuals from lower socioeconomic position (SEP) also spent more time in their neighbourhood, thereby being more exposed to the food environment around home [16]. Although this may possibly result in a differential impact of the food environment on overweight and obesity across socioeconomic groups, a recent systematic review of mostly cross-sectional studies could not confirm this [17]. Longitudinal studies focussing on socioeconomic inequalities in the food environment and related changes in obesity are needed.

The Generation R Study is a birth-cohort study in which four objective measures of BMI and two indices of fat mass obtained from dual-energy x-ray absorptiometry (DXA) scanners were available for children between the age of 4 and 14 years. Linkage with yearly updated food environment measures created the unique possibility to evaluate whether changes in the food environment were associated with changes in body composition. We hypothesised that children with parents of lower SEP lived in neighbourhoods characterised by a higher number of unhealthy food outlets, and that over time, this resulted in unfavourable changes in body composition. First, we evaluated if the exposure to food environment around the home address differentially evolved over time for children from lower and higher educated mothers using up to four repeated measures between the age of 4 and 14 years. Second, we studied if the impact of changes in the food environment on changes in measures of body composition differed between children from lower and higher educated mothers.

## Materials and methods

### Study design

This study used data collected from the Generation R Study, a prospective birth-cohort study in the city of Rotterdam, in The Netherlands [18]. We included objectively determined measures of BMI at the age of 4, 6, 10 and 14 years, and DXA-derived measures of fat mass at the age of 6 and 10 years. At each time-point, home addresses were linked with food environment data of the preceding year. The latter were only available from 2004 onwards, therefore we did not include outcomes collected in preceding years.

### Study population

Invitations to participate in the Generation R Study were sent out to all pregnant women who had an expected delivery date between April 2002 and January 2006 and who lived in the study area (Rotterdam, The Netherlands) at time of delivery [18]. The Medical Ethics Committee of the Erasmus University Medical Centre in Rotterdam approved the study (MEC 217.595/2002/20). Written informed consent was obtained from parents at child ages of 4, 6 and 10 years, and from parents and children at the age of 14 years.

**Fig. 1:**
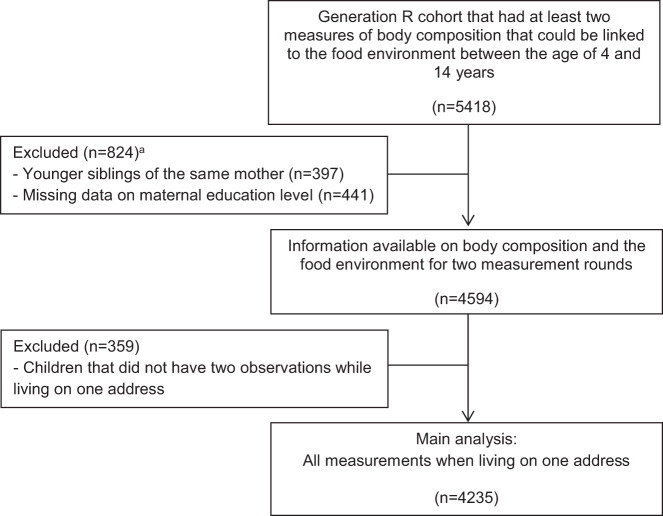
Flow diagram. ^a^More than one reason could apply.

In total, 9901 children and their parents participated in the Generation R Study at baseline. Children with at least two outcome measures that could be linked to the food environment between the ages of 4 and 14 years (*n* = 5418) were included. Younger siblings from the same mother (*n* = 397), and children from which information on maternal education level were missing (*n* = 441) were excluded. In total, 4594 children were eligible for the present study. We included observations for the address where the child lived for the longest period between the age of 4 and 14 years, and children without two consecutive observations on one address (*n* = 359) were excluded. The sample for the main analysis included 4235 children with 11,277 person-observations for BMI, and 6240 person-observations for fat mass (Fig. 1).

### Food environment

Data on location (*X* and *Y* coordinates) and type of food outlet were obtained from Locatus, a commercial company that collects information on food retailers in The Netherlands by means of yearly field audits. A validation study using field audit data found an overall ‘good’ to ‘excellent’ agreement for both the location and classification [19]. *X* and *Y* coordinates of children’s home addresses, and *X* and *Y* coordinates of food retailers in the year preceding the outcome measure, were mapped using a geographic information system (GIS) (GIS-ArcGIS 10, ESRI, Redlands, CA, USA). Euclidean buffers of 400 m were used to count the number of food retailers located around the home, for each time-point in the study. The 400 m distance represents a walking distance of ~5 min. Dutch cities are characterised by high connectivity that is supportive of active forms of transportation. Therefore, a distance of 400 m seems an appropriate distance to assess the food environment in this context.

We considered all fast-food outlets, grillrooms/kebab shops, take away outlets, and ice cream shops that were classified by Locatus and available in the database as ‘fast-food outlets’. The ‘absolute fast-food exposure’ reflects the total number of fast-food outlets within the 400 m buffer around home. Because the dominance of fast-food outlets over other types of food outlets may drive purchasing behaviour and diet quality [13, 20], we also calculated the ‘relative fast-food exposure’ by dividing the total number of fast-food outlets by the total number of food outlets.

The food environment consists of a variety of outlets that may all influence food choices and ultimately obesity. To capture the healthiness of the food environment, we also calculated a healthiness score of all food outlets previously developed in a Delphi study [21]. Scores ranged from −5 points (very unhealthy) to +5 points (very healthy). Fast-food was considered the most unhealthy food outlet (−4.9 points), whereas the green-grocer was considered the most healthy food outlet (4.8 points). We calculated the average healthiness score of the food environment within 400 m from home. Addresses without any food outlet were excluded, since it is uncertain what value to assign to these addresses.

### Body composition measures

At the age 4 years, body height and weight measurements were performed during routine visits at the Child Health Centres. At the age of 6, 10 and 14 years, children visited the research centre in the Erasmus Medical Centre for detailed physical examinations. During all examinations, height and weight were measured without shoes and heavy clothing. Height was measured to the nearest millimetre by a stadiometer (Holtain Limited, Dyfeld, UK). Weight was measured to the nearest gram using an electronic scale (SECA, Almere, The Netherlands). BMI was calculated [weight (kg)/height (m^2^)] and age- and sex-specific standard deviation scores (SDS) for BMI were obtained from Dutch reference growth charts (Growth Analyzer 4.0, Dutch Growth Research Foundation) [22].

Body composition was measured at the research centre at the age of 6 and 10 years using a DXA scanner (iDXA, GE-Lunar, 2008, Madison, WI, USA) using enCORE software version 12.6. Children were placed without shoes, heavy clothing and metal objects in supine position on the DXA table. We calculated fat mass index (FMI) [fat mass (kg)/height (m^3^)] and fat-free mass index (FFMI) [fat-free mass (kg)/height (m^3^)] according to standard procedures [23]. We calculated SDS for FMI and FFMI [(observed value − mean)/SD] on the basis of the cross-sectional sample distribution within the Generation R Study population to enable comparisons of effect estimates for FMI and FFMI.

### Maternal education level

We used maternal education level to stratify the analyses by SEP. The highest education level attained was established by questionnaire at the child’s age of 6 years, and categorized according to the Dutch Standard Classification of Education into high (university degree), mid-high (higher vocational training, bachelor’s degree), mid-low (>3 years general secondary school, intermediate vocational training) and low (no education, primary school, lower vocational training, intermediate general school or ≤3 years general secondary school)) [24].

### Other sociodemographic variables

Sociodemographic characteristics obtained at baseline and during follow-up visits by means of questionnaires included age, sex, ethnicity and net household income. In accordance with Statistics Netherlands [25], a child’s ethnic background was classified as native Dutch, other-Western background, and non-Western background based on the country of birth of the child’s parents. Net household income was asked for at the child’s age of 6, 10 and 14 years, and categorized into low (≤€2000/month), intermediate (<€2000–€3200/month and <€2000–€3300/month at the age of 14 years) and high (>€3200/month and >€3300/month at the age of 14 years).

### Statistical analyses

Characteristics of the children during the first and last measurement when living on the same address were presented. Number of missing values were presented in the supplement (Appendix 1). The median follow-up period was 7.1 years. Yet, large differences were seen across levels of maternal education, with children from lower educated mothers having the shortest follow-up time. Therefore, changes in the food environment over time were standardised, and expressed as the changes over 7.1 years. Kernel density plots with normal distribution approximation [26] were used to visualise the within-person changes in the food environment over time. Histograms suggested that the change over time approximated a normal distribution (Appendix 2). Linear regression models were used to test the change in the food environment over 7.1 years by four levels of maternal education. Associations were expressed as the change in relative contribution of fast-food outlets, absolute number of fast-food outlets, and the healthiness of the food environment over 7.1 years.

Fixed-effects linear regression models with repeated measurements were used to study within-person changes in the food environment and the association with within-person changes in body composition measures. We tested for interactions with maternal education level to assess whether the association between changes in the food environment and changes in body composition differed by level of maternal education. To retain statistical power, maternal education was categorised into lower (low, mid-low) and higher (mid-high, high) education. We used up to four measures of BMI, and two measures of FMI and FFMI. This method allowed to control for measured time-variant variables and for unmeasured time-invariant variables [27]. All models were specified using the first-difference model, and evaluated the change between two waves available, which were not necessarily two consecutive waves. Models were adjusted for the time between measurements. The specification of the model is presented in the supplement (Appendix 3).

Results were presented as a relative increase by 10%-point in fast-food outlets, an absolute increase by one fast-food outlet, and per 0.5 point increase in healthiness score of the food environment (which indicates a healthier environment). Units were based on the average change for those with changes in the population between time-points (Supplementary Table 1).

The aforementioned analyses assume a linear exposure–response relationship, whereby every unit decrease or increase in the food environment has a similar effect. However, changes observed for children without any fast-food outlet available (e.g. from 0 to 2 outlets) may have stronger effects than similar changes observed at the higher end of the distribution (e.g. from 5 to 7 outlets). Hence, we also performed analyses restricted to children without any fast-food outlets during the first measurement round, and explored if the introduction of fast-food outlets mattered for the subsequent changes in outcomes.

Two sensitivity analyses were conducted. First, we controlled for the time-varying factor net household income, since having a higher income may contribute to healthier dietary behaviours [28]. Income was not collected at age 4 years, thus this analysis was restricted for the age of 6, 10 and 14 years. We excluded children for which net household income was missing at all time-points (*n* = 222). Income did not change between time-points for 77.5% of the children. We imputed income measured at the nearest time-point assuming that income did not change (12.7% imputations). Second, we additionally included changes resulting from residential moves. In this analyses, we included 4594 children with 13,528 person-observations of BMI, and 7856 person-observations on fat mass.

All analyses were conducted in R version 3.4.1, using the plm package for the fixed-effects analyses. Clustered sandwich estimators were used to allow for within-child correlation between error terms. Two-sided *P* values <0.05 were considered statistically significant. R scripts are available for researchers upon authors request.

## Results

**Fig. 2:**
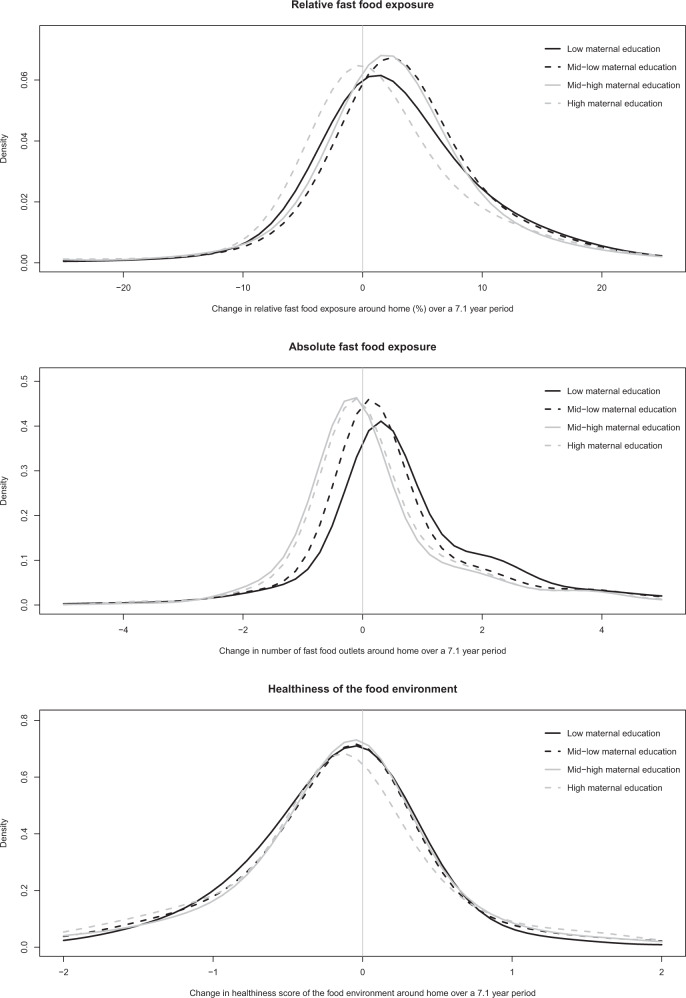
Changes in the food environment around home over a 7.1-year period by maternal education level. Presented are Kernel density plots. The bandwidth was based on Silverman’s rule of thumb, the normal distribution approximation. Bandwidth was set equal for all groups, using the mean of the normal optimal values for the four different groups by maternal education level.

At all time-points, children from lower educated mothers were exposed to more fast-food outlets, for both the relative and absolute exposure to fast-food outlets. These children were also exposed to unhealthier food outlets, and had a higher BMI and FMI than children from higher educated mothers (Table 1). Some children were exposed to a healthier food environment over time, but a larger proportion of children were exposed to an unhealthier food environment (Fig. 2). Further, the relative and absolute change in exposure to fast-food outlets over 7.1 years increased more rapidly for children from low as compared to high educated mothers (2.0%-point (95% CI: 0.7–3.4) and 0.6 fast-food outlet (95% CI: 0.4–0.8), respectively). The change in healthiness of the food environment over time did not differ across the four levels of maternal education.

**Table 1:** Characteristics of children from the Generation R Study.

	First measurement				Last measurement			
	Low (*n* = 838)	Mid-low (*n* = 1315)	Mid-high (*n* = 944)	High (*n* = 1138)	Low (*n* = 838)	Mid-low (*n* = 1315)	Mid-high (*n* = 944)	High (*n* = 1138)
**Child characteristics**								
Age, year	6.5 ± 1.9^a^	6.3 ± 1.8	6.3 ± 1.7	6.3 ± 1.9	12.0 ± 2.3	12.3 ± 2.2	12.5 ± 1.9	12.6 ± 2.0
Girls, *n* (%)	424 (50.6)	661 (50.3)	460 (48.7)	573 (50.4)	424 (50.6)	661 (50.3)	460 (48.7)	573 (50.4)
Ethnicity, *n* (%)								
Dutch	315 (37.6)	712 (54.1)	668 (70.8)	872 (76.6)	315 (37.6)	712 (54.1)	668 (70.8)	872 (76.6)
Non-Western	480 (57.3)	504 (38.3)	169 (17.9)	122 (10.7)	480 (57.3)	504 (38.3)	169 (17.9)	122 (10.7)
Other-Western	43 (5.1)	99 (7.5)	106 (11.2)	144 (12.7)	43 (5.1)	99 (7.5)	106 (11.2)	144 (12.7)
Net household income, *n* (%)								
Low	268 (32.0)	241 (18.3)	67 (7.1)	33 (2.9)	265 (31.6)	261 (19.8)	68 (7.2)	34 (3.0)
Intermediate	142 (16.9)	327 (24.9)	190 (20.1)	89 (7.8)	198 (23.6)	324 (24.6)	218 (23.1)	146 (12.8)
High	77 (9.2)	337 (25.6)	461 (48.8)	717 (63.0)	98 (11.7)	457 (34.8)	518 (54.9)	845 (74.3)
**Measures of body composition**^b^								
BMI SDS	0.46 ± 1.15	0.24 ± 0.97	0.12 ± 0.87	0.07 ± 0.80	0.71 ± 1.20	0.37 ± 1.15	0.06 ± 1.01	−0.01 ± 0.99
FMI SDS	0.37 ± 1.38	0.03 ± 1.07	−0.20 ± 0.91	−0.35 ± 0.73	0.64 ± 1.75	0.15 ± 1.47	−0.30 ± 1.18	−0.50 ± 0.96
FFMI SDS	0.05 ± 0.87	−0.04 ± 0.82	−0.02 ± 0.80	−0.01 ± 0.76	0.18 ± 0.86	0.00 ± 0.80	−0.07 ± 0.74	−0.11 ± 0.72
**Food environment within 400** **m around home**								
Total food outlets, *n*	16 [3, 41]^c^	7 [1, 25]	5 [1, 20]	6 [1, 20]	16 [3, 40]	7 [1, 24]	5 [1, 20]	5 [1, 20]
Fast-food outlets, *n*	2 [0, 7]	1 [0, 4]	1 [0, 3]	1 [0, 3]	3 [1, 7]	1 [0, 5]	1 [0, 4]	1 [0, 3]
Relative fast-food (%)	18 ± 16	16 ± 16	15 ± 16	16 ± 19	21 ± 18	20 ± 18	17 ± 17	19 ± 19
Healthiness score	−0.86 ± 1.15	−0.69 ± 1.17	−0.62 ± 1.15	−0.69 ± 1.27	−0.98 ± 1.20	−0.84 ± 1.24	−0.76 ± 1.20	−0.81 ± 1.20

Number of missing values are presented in the Appendix 1.

^a^Values are means ± SD (all such values).

^b^FMI and FFMI were only available at age 6 and 10 years.

^c^Values are medians [IQR] (all such values).

*BMI* body mass index, *FMI* fat-mass index, *FFMI* fat-free mass index.

At their first measurement round, 39.7% of the children from low educated mothers and 20.7% of the children from high educated mothers were exposed to four or more fast-food outlets in the direct vicinity of their home. Subsequent changes in fast-food outlets, both in relative and absolute terms, and the healthiness of the food environment were not associated with changes in body composition (Table 2). Small increases in FMI SDS were seen for children from lower educated mothers with a higher relative fast-food exposure over time, and this was not observed for children from higher educated mothers.

**Table 2:** Association between changes in the food environment and changes in body composition measures.

	Lower maternal education level			Higher maternal education level		
	Persons	Person-observations	Estimate (95% CI)	Persons	Person-observations	Estimate (95% CI)
Relative fast-food exposure (+10%-point)						
BMI (SDS)	2153	5605	0.01 (−0.01; 0.04)	2082	5672	0.01 (−0.01; 0.02)
FMI (SDS)	1561	3122	**0.04 (0.00; 0.08)**	1559	3118	0.01 (−0.01; 0.04)
FFMI (SDS)	1561	3122	0.00 (−0.02; 0.03)	1559	3118	0.01 (−0.01; 0.03)
Absolute fast-food exposure (+1 outlet)						
BMI (SDS)	2153	5605	0.01 (−0.01; 0.02)	2082	5672	0.01 (−0.01; 0.02)
FMI (SDS)	1561	3122	0.02 (−0.00; 0.04)	1559	3118	0.01 (−0.01; 0.04)
FFMI (SDS)	1561	3122	0.01 (−0.01; 0.02)	1559	3118	−0.00 (−0.02; 0.01)
Healthiness score (+0.5 point)						
BMI (SDS)	1812	4558	0.00 (−0.02; 0.02)	1657	4363	0.01 (−0.01; 0.02)
FMI (SDS)	1223	2446	−0.01 (−0.03; 0.02)	1170	2340	0.00 (−0.02; 0.02)
FFMI (SDS)	1223	2446	−0.01 (−0.02; 0.01)	1170	2340	−0.00 (−0.02; 0.01)

Estimates were obtained from fixed-effects linear regression using a first-difference model specification. All analyses were adjusted for the time between measurements. BMI was collected at age 4, 6, 10 and 14 years, FMI and FFMI at age 6 and 10 years.

Bold values indicate statistical significance.

*BMI* body mass index, *FMI* fat-mass index, *FFMI* fat-free mass index.

When focussing on children without fast-food around home during the first measurement round, small increases in BMI were observed for children from lower educated mothers in which relative and absolute fast-food exposure increased (Table 3). Primarily increases in FMI were observed, however, confidence intervals were wide. For children from higher educated mothers without fast-food outlets at first, the introduction of fast-food was not associated with body composition measures.

**Table 3:** Associations between changes in the food environment and changes in body composition measures for children without fast-food outlets around home during the first measurement round.

	Lower maternal education level			Higher maternal education level		
	Persons	Person-observations	Estimate (95% CI)	Persons	Person-observations	Estimate (95% CI)
Relative fast-food exposure (+10%-point)						
BMI (SDS)	705	1879	**0.04 (0.00; 0.07)**	914	2468	−0.02 (−0.04; 0.00)
FMI (SDS)	546	1092	0.07 (−0.02; 0.16)	671	1342	0.01 (−0.03; 0.04)
FFMI (SDS)	546	1092	0.03 (−0.02; 0.07)	671	1342	−0.00 (−0.02; 0.02)
Absolute fast-food exposure (+1 outlet)						
BMI (SDS)	705	1879	0.06 (−0.00; 0.12)	914	2468	−0.04 (−0.12; 0.03)
FMI (SDS)	546	1092	0.09 (−0.03; 0.20)	671	1342	−0.08 (−0.20; 0.02)
FFMI (SDS)	546	1092	−0.00 (−0.05; 0.05)	671	1342	−0.02 (−0.07; 0.03)

Estimates were obtained from fixed-effects linear regression using a first-difference model specification. All analyses were adjusted for the time between measurements. BMI was collected at age 4, 6, 10 and 14 years, FMI and FFMI at age 6 and 10 years.

Bold values indicate statistical significance.

*BMI* body mass index, *FMI* fat-mass index, *FFMI* fat-free mass index.

Additionally adjusting for the time-varying factor net household income did not change the findings (Supplementary Table 2a, b). None of the associations found in the main analyses were found when studying changes over time when additionally including changes resulting from residential moves (Supplementary Table 3a, b).

## Discussion

Children from lower educated mothers had more fast-food outlets as well as an unhealthier food environment around home. These children were also at higher risk that the food environment unfavourably changed offering more fast-food and other unhealthy food outlets. We observed that 39.7% of the children from low educated mothers were exposed to four or more fast-food outlets, whereas this was 20.7% of the children from higher educated mothers. Subsequent changes in fast-food outlets or in the healthiness of the food environment were not associated with body composition measures. For children from lower educated mothers without fast-food exposure during the first measurement round, small increases in BMI were seen following the introduction of fast-food outlets.

### Strengths and limitations

A strength of the study was the use of repeated measures of the food environment and objective measures of body composition among a large cohort of school-aged children. A 2–4-year difference between time-points was sufficiently large so that a group of children experienced changes in the food environment around home, but small enough to be able to attribute the change in body composition to changes in the food environment. Some of the bias that may have arisen from residential self-selection was taken into account by using a fixed-effects approach. Focusing on children in which the exposure of food outlets around home changed during follow-up, as opposed to cross-sectional studies and studies in which children move across neighbourhoods, is essential for this purpose.

Our study also had some weaknesses. First, we aimed to approximate causal effects by evaluating changes over time in the food environment, but time-variant variables that we were unable to control for may have caused confounding. These could include factors related to dietary choices (e.g. preference for fast-food), but also factors influencing body composition (e.g. financial stress) occurring for children that also experienced changes in the food environment. Instrumental variable (IV) approaches may further eliminate bias from unobserved time-varying confounding, and two studies found that associations between the food environment and diet quality or BMI were stronger in magnitude using IV models compared to fixed-effects analyses [13, 14]. Identifying a valid instrument to study built environment changes is challenging, and this was not further explored in the current study. Second, perceived exposure and access to fast-food outlets may yield more consistent findings, but we lacked this information [6, 8, 29]. Third, we were not able to evaluate changes occurring at other locations where children and their parents were exposed to food (e.g. school environment), or changes made to the physical activity environment (e.g. new playgrounds) that may also affect body composition. Assessments of the food environment based on addresses, rather than using administrative boundaries of neighbourhoods, may have prevented some of the biases resulting from changes occurring in the neighbourhood. Finally, the current evaluation lacked insights into the mechanism through which the food environment affect purchasing behaviours, dietary patterns and subsequently body composition.

The current evidence base defined and observed inequalities in the fast-food environment in different ways. A review reported that fast-food locations were more prevalent in low-income neighbourhoods and neighbourhoods with high concentrations of ethnic minority groups [15]. Although conceptualised differently, they point to similar conclusions that exposure to the food environment is not equally distributed across the population. Furthermore, we found that the food environment shifted towards a higher exposure of unhealthy foods, and this unhealthy shift was more likely to occur for children from lower educated mothers. Similar patterns were observed in a study conducted in New York City, US, suggesting that the number of unhealthy food outlets increased more rapidly in neighbourhoods with a higher initial number of unhealthy food outlets, and for disadvantaged neighbourhoods [30].

The counterintuitive finding of a US study, suggesting that BMI decreased following the increase in fast-food outlets, was not found in our study [10]. No changes in body composition were observed following changes in the food environment in the total population. A likely explanation is that the introduction of one additional fast-food outlet or changes in healthiness of food outlets were too small to have a noticeable effect on obesity measures for children living in a so-called ‘fast-food paradise’, given the numerous food outlets around home. Importantly, the entire population is exposed to small shifts in the food environment. Even small changes in the distribution may therefore have a large impact on disease risk on the level of the population. These shifts are also important because they are thought to contribute to socioeconomic inequalities in health; shifting the curve by universal prevention strategies is considered to be an important strategy in reducing the health disparities [31].

Our results gave some indications that fast-food exposure was associated with small increases in BMI for children of lower educated mothers that were not exposed to fast-food during the first measurement round, but not for children from higher educated mothers. DXA scans suggested that especially fat mass increased following the introduction of fast-food outlets. We are not aware of any other study looking at changes in fat mass in relation to the built environment. In sensitivity analyses, no associations between the food environment and body composition were found when additionally including changes resulting from residential moves. This could suggest that other factors that change during residential relocation, such as the impact of changing schools, may contribute to body composition to a larger degree.

Other longitudinal studies using a fixed-effects approach reported that fast-food outlets were associated with higher fast-food intake among young adults from low-income families, but not among higher-income families [11]. Another study found that favourable changes in the food environment were related to BMI reductions in adults with obesity, but not for normal weight or overweight adults [12]. Furthermore, a review based on primarily cross-sectional studies reported that a higher fast-food exposure was associated with obesity for children from lower-income households or neighbourhoods [9]. These findings show the importance to thoroughly examine differential effects of the food environment on dietary behaviours and body composition outcomes.

The high and increasing number of fast-food outlets, and the larger exposure to them of children from lower socioeconomic groups provide support to policies aimed at ‘shifting the curve’. To date, strategies to improve dietary habits mainly rely on nutritional education rather than on structural interventions to modify the food environment. That changes in BMI were small following the introduction of fast-food outlets around home between a 2- and 4-year period should not limit efforts to do so. The cumulative effect of the food environment over time and across various daily activity settings was not explored, but may steadily impact on children’s body composition. Unravelling causal mechanisms how fast-food outlets influences body composition, for example by having information on fast-food consumption or dietary patterns, is essential. Furthermore, the wider context in which food environments change and food choices are made is important to consider. It has been suggested that more food outlets could reflect general investments made to that area, which could positively or negatively influence children’s body composition [30]. More insights are needed to understand these type of complexities around childhood obesity [32].

In conclusion, this study provided evidence of widening socioeconomic inequalities in the food environment around children’s homes growing up in a large urban area in The Netherlands over a period of 7.1 years. Children from lower educated mothers were at higher risk that the food environment shifted more rapidly towards an unhealthy environment. Exposure to more fast-food outlets does not seem to have an additional impact on BMI in contemporary contexts with ubiquitous fast-food outlets. These findings support the importance of an equity-focus on the link between the food environment and body composition.

## Supplementary information

Supplemental material

## Data Availability

The dataset generated for the present study is not publicly available, as participants’ consent was not obtained for data sharing. R scripts are available upon request from the authors.
