# Supplementary material for: Socioeconomic inequalities in the food environment and body composition among school-aged children: a fixed-effects analysis
Source: Int J Obes (Lond). 2021 Aug 13;45(12):2554–61. doi: 10.1038/s41366-021-00934-y (PMC8606311; doi:10.1038/s41366-021-00934-y)
Supplement: Supplementary file 1 — Supplemental material [file 41366_2021_934_MOESM1_ESM.docx]

**Online Supplemental Material**

**Socioeconomic inequalities in the food environment and body composition among school-aged children: a fixed-effects analysis**

Famke J.M. Mölenberg, Joreintje D. Mackenbach, Maartje P. Poelman, Susana Santos, Alex Burdorf, Frank J. van Lenthe

**Contents**

Appendix 1 Number of missings

Appendix 2 Histograms change in the food environment over 7.1 year period

Appendix 3 Model specification

Supplemental Table 1 Change in the food environment between time-points

Supplemental Table 2a Associations between changes in the food environment and changes in body composition measures adjusted for income

Supplemental Table 2b Associations between changes in the food environment and changes in body composition measures for children without fast food outlets around home, adjusted for income

Supplemental Table 3a Associations between changes in the food environment and changes in body composition, including children that moved houses

Supplemental Table 3b Associations between changes in the food environment and changes in body composition for children without fast food outlets around home, including children that moved houses

**Appendix 1 – Number of missings**

|  | First measurement round | | | |
| --- | --- | --- | --- | --- |
|  | Low  (n=838) | Mid-low (n=1315) | Mid-high (n=944) | High (n=1138) |
| Age | 0 (0.0) | 0 (0.0) | 0 (0.0) | 0 (0.0) |
| Sex | 0 (0.0) | 0 (0.0) | 0 (0.0) | 0 (0.0) |
| Ethnicity | 0 (0.0) | 0 (0.0) | 1 (0.1) | 0 (0.0) |
| Net household income | 351 (41.9) | 410 (31.2) | 226 (23.9) | 299 (26.3) |
| BMI | 0 (0.0) | 0 (0.0) | 0 (0.0) | 0 (0.0) |
| FMI | 53 (6.3) | 72 (5.5) | 53 (5.6) | 85 (7.5) |
| FFMI | 53 (6.3) | 72 (5.5) | 53 (5.6) | 85 (7.5) |

|  | Last measurement round | | | |
| --- | --- | --- | --- | --- |
|  | Low  (n=838) | Mid-low (n=1315) | Mid-high (n=944) | High (n=1138) |
| Age | 0 (0.0) | 0 (0.0) | 0 (0.0) | 0 (0.0) |
| Sex | 0 (0.0) | 0 (0.0) | 0 (0.0) | 0 (0.0) |
| Ethnicity | 0 (0.0) | 0 (0.0) | 1 (0.1) | 0 (0.0) |
| Net household income | 277 (33.1) | 273 (20.8) | 140 (14.8) | 113 (9.9) |
| BMI | 0 (0.0) | 0 (0.0) | 0 (0.0) | 0 (0.0) |
| FMI | 83 (9.9) | 100 (7.6) | 46 (4.9) | 69 (6.1) |
| FFMI | 83 (9.9) | 100 (7.6) | 46 (4.9) | 69 (6.1) |

**Appendix 2 – Histograms change in the food environment over 7.1 year period**


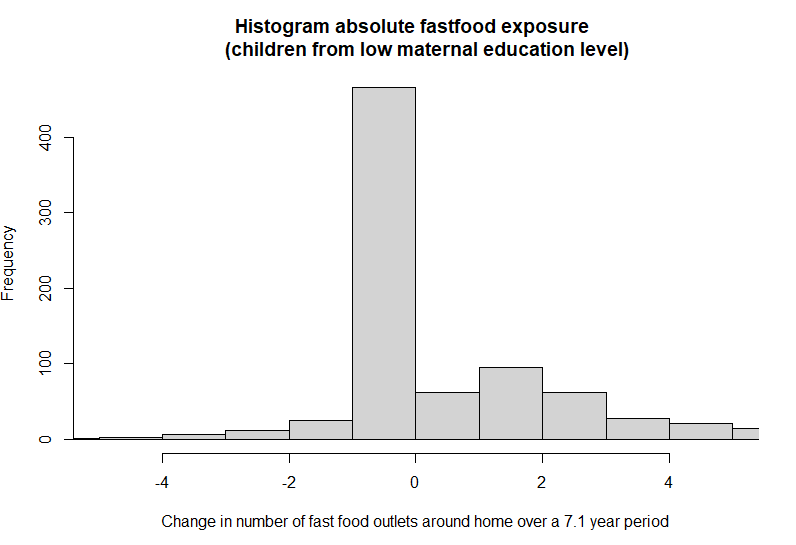

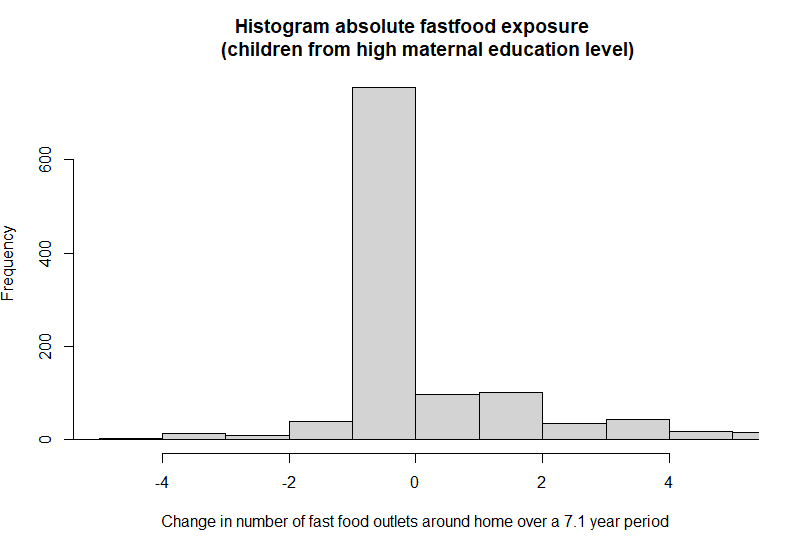

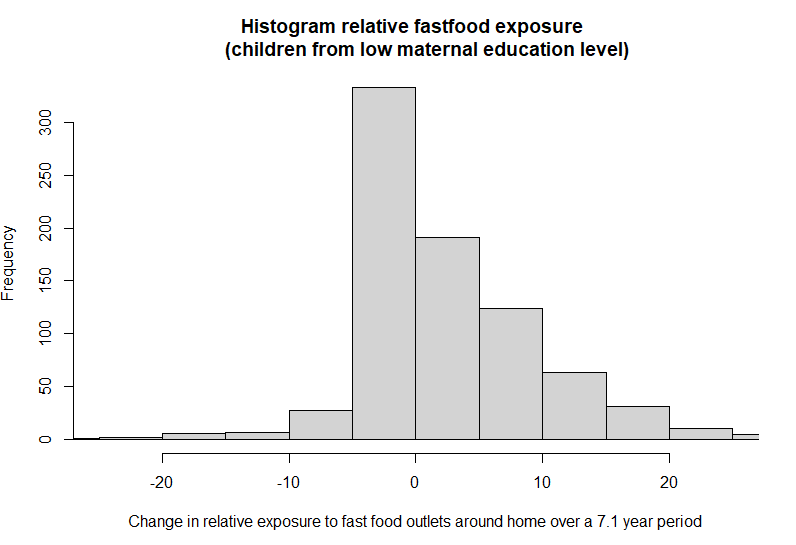

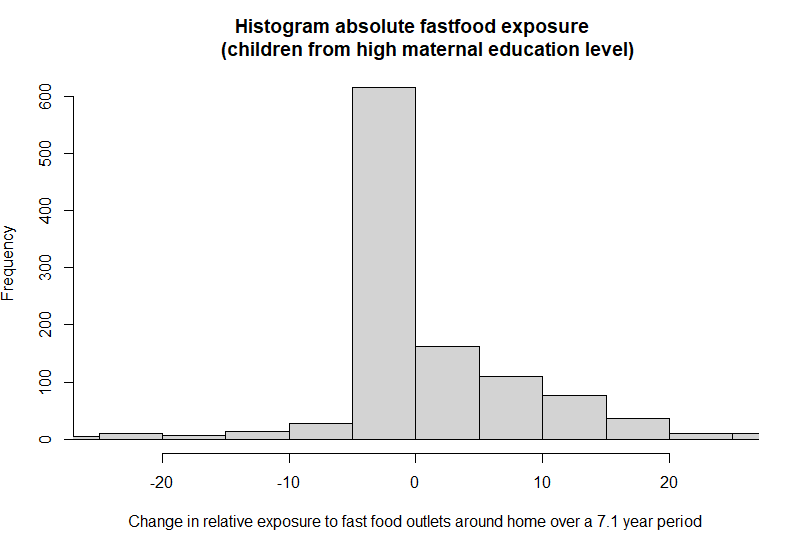


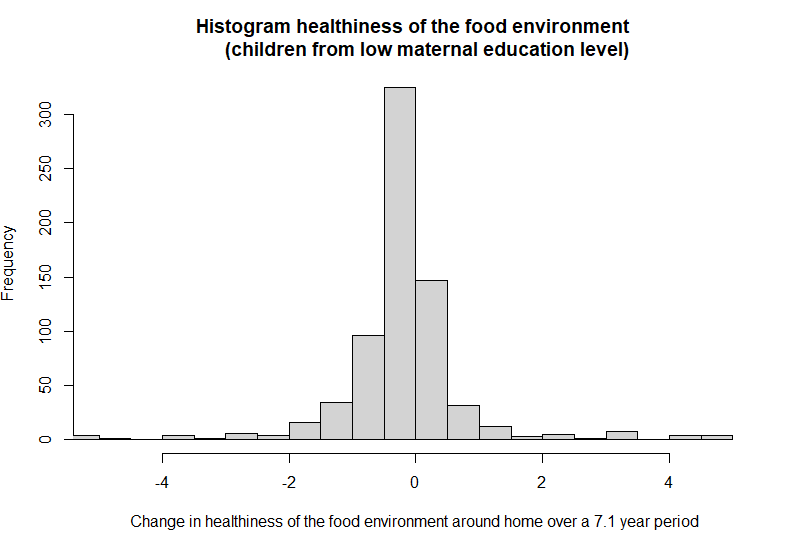

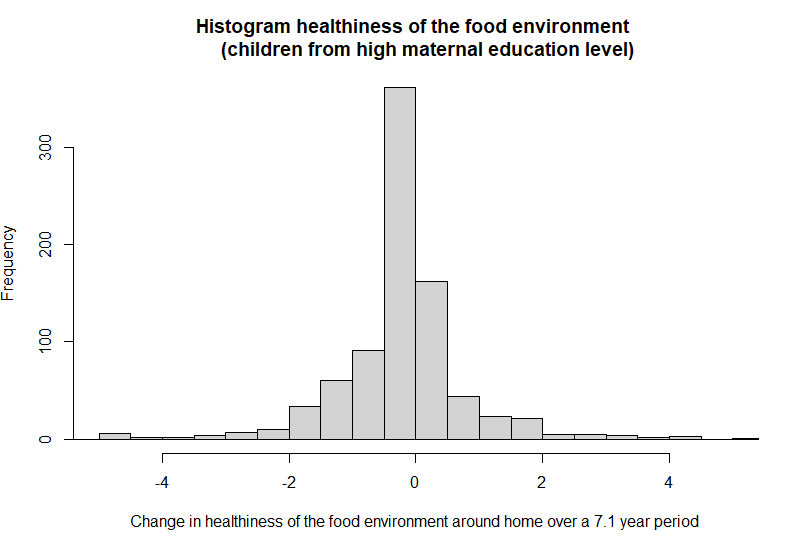


**Appendix 3 – Model specification**

The following regression model was used:

$$y_{it-1}= \alpha_{t}+{\beta_{1} (food environment}_{it-1})+ {\beta_{2} (x}_{it-1})+\mu_{i}+ \varepsilon_{it-1}$$

Where $y_{\mathrm{it}}$ is the dependent variable of interest (e.g. BMI SDS, FMI SDS, FFMI SDS) for individual i at time t, $\beta_{1}$ is the effect of changes in the food environment for individual i on time t-1 (e.g. absolute fast food exposure, relative fast food exposure, healthiness score of the food environment), $\beta_{2}$ is the effect of time-varying factors for individual i on time t-1, $\alpha_{t}$ accounts for time effects that are constant across individuals,$\mu_{i}$ accounts for time-invariant random errors on the individual-level, and $\varepsilon_{\mathrm{it}}$ accounts for normal sources of error that vary across individuals and time.

Supplemental Table 1: Change in the food environment between time-points

|  | |  | | Lower maternal education level (n=2153) | | | | | Higher maternal education level (n=2082) | | | | |
| --- | --- | --- | --- | --- | --- | --- | --- | --- | --- | --- | --- | --- | --- |
|  | |  |  | Persons | | Mean difference (SD) | | | Persons | | Mean difference (SD) | | |
| Relative fast food exposure (%) | | | | | | | | |  | |  | | |
| 4-6 year | | | | | | | | |  | |  | | |
| Decrease | |  |  | 78 | | -2.6 (3.5) | | | 53 | | -3.8 (7.6) | | |
| Increase | |  |  | 90 | | 3.4 (6.4) | | | 80 | | 4.8 (8.5) | | |
| No change | |  |  | 211 | | 0 (0) | | | 245 | | 0 (0) | | |
| 6-10 year | | | | | | | | | |  | | | |
| Decrease | |  |  | 214 | | -9.1 (12.9) | | | 256 | | -9.2 (12.3) | | |
| Increase | |  |  | 689 | | 7.0 (9.9) | | | 584 | | 7.5 (10.3) | | |
| No change | |  |  | 707 | | 0 (0) | | | 779 | | 0 (0) | | |
| 10-14 year | | | | | | | | | |  | | | |
| Decrease | |  |  | 249 | | -6.9 (12.3) | | | 253 | | -10.4 (17.9) | | |
| Increase | |  |  | 540 | | 7.7 (12.4) | | | 496 | | 7.3 (12.3) | | |
| No change | |  |  | 596 | | 0 (0) | | | 789 | | 0 (0) | | |
| Absolute fast food exposure | | | | | | | |  | | | |  | |
| 4-6 year | | | | | | | |  | | | |  | |
| Decrease |  | |  | | 34 | | -1.2 (0.5) | | 20 | | | | -1.3 (0.6) |
| Increase |  | |  | | 41 | | 1.1 (0.3) | | 47 | | | | 1.1 (0.5) |
| No change |  | |  | | 304 | | 0 (0) | | 311 | | | | 0 (0) |
| 6-10 year | | | | | | | | | |  | | | |
| Decrease |  | |  | | 164 | | -1.4 (0.7) | | 195 | | | | -1.2 (0.5) |
| Increase |  | |  | | 456 | | 2.1 (1.8) | | 399 | | | | 1.6 (1.1) |
| No change |  | |  | | 990 | | 0 (0) | | 1025 | | | | 0 (0) |
| 10-14 year | | | | | | | | | |  | | | |
| Decrease |  | |  | | 131 | | -1.3 (0.6) | | 134 | | | | -1.3 (0.6) |
| Increase |  | |  | | 405 | | 2.2 (2.0) | | 345 | | | | 2.1 (1.9) |
| No change |  | |  | | 849 | | 0 (0) | | 1059 | | | | 0 (0) |
| Healthiness score | | | | | | | | |  | |  | | |
| 4-6 year | | | | | | | | |  | |  | | |
| Decrease (unhealthier) | |  |  | 87 | | -0.4 (0.6) | | | 91 | | -0.5 (0.7) | | |
| Increase (healthier) | |  |  | 98 | | 0.2 (0.3) | | | 60 | | 0.3 (0.5) | | |
| No change | |  |  | 131 | | 0 (0) | | | 162 | | 0 (0) | | |
| 6-10 year | | | | | | | | | |  | | | |
| Decrease (unhealthier) | |  |  | 599 | | -0.5 (0.7) | | | 534 | | -0.6 (0.8) | | |
| Increase (healthier) | |  |  | 394 | | 0.4 (0.6) | | | 403 | | 0.4 (0.5) | | |
| No change | |  |  | 270 | | 0 (0) | | | 280 | | 0 (0) | | |
| 10-14 year | | | | | | | | | |  | | | |
| Decrease (unhealthier) | |  |  | 554 | | -0.5 (0.5) | | | 511 | | -0.5 (0.6) | | |
| Increase (healthier) | |  |  | 280 | | 0.5 (0.8) | | | 307 | | 0.6 (0.7) | | |
| No change | |  |  | 266 | | 0 (0) | | | 316 | | 0 (0) | | |

Supplemental Table 2a: Associations between changes in the food environment and changes in body composition measures adjusted for income

|  | Lower maternal education level | | | Higher maternal education level | | |
| --- | --- | --- | --- | --- | --- | --- |
|  | Persons | Person-observations | Estimate  (95% CI) | Persons | Person-observations | Estimate  (95% CI) |
| Relative fast food exposure (+10%-point) |  |  |  |  |  |  |
| BMI (SDS) | 1973 | 4841 | 0.01 (-0.01; 0.04) | 2040 | 5185 | 0.01 (-0.01; 0.02) |
| FMI (SDS) | 1456 | 2912 | **0.04 (0.00; 0.08)** | 1532 | 3064 | 0.01 (-0.01; 0.03) |
| FFMI (SDS) | 1456 | 2912 | 0.00 (-0.02; 0.03) | 1532 | 3064 | 0.01 (-0.01; 0.03) |
| Absolute fast food exposure (+1 outlet) |  |  |  |  |  |  |
| BMI (SDS) | 1973 | 4841 | 0.01 (-0.00; 0.03) | 2040 | 5185 | 0.00 (-0.02; 0.02) |
| FMI (SDS) | 1456 | 2912 | 0.02 (-0.00; 0.05) | 1532 | 3064 | 0.01 (-0.02; 0.03) |
| FFMI (SDS) | 1456 | 2912 | 0.01 (-0.00; 0.02) | 1532 | 3064 | -0.00 (-0.02; 0.01) |
| Healthiness score  (+0.5 point) |  |  |  |  |  |  |
| BMI (SDS) | 1649 | 3909 | -0.00 (-0.02; 0.02) | 1621 | 3958 | 0.01 (-0.01; 0.02) |
| FMI (SDS) | 1139 | 2278 | -0.01 (-0.04; 0.02) | 1149 | 2298 | 0.00 (-0.02; 0.02) |
| FFMI (SDS) | 1139 | 2278 | -0.02 (-0.03; 0.01) | 1149 | 2298 | -0.00 (-0.02; 0.01) |

Estimates were obtained from fixed-effects linear regression using a first-difference model specification. All analyses were adjusted for the time between measurements and net household income. Income was not collected at age 4 years, thus this analyses was restricted for the age of 6, 10, and 14 years. Missing income was imputed using income measured at the nearest time-point. BMI was collected at age 6, 10 and 14 years, FMI and FFMI at age 6 and 10 years.

Supplemental Table 2b: Associations between changes in the food environment and changes in body composition measures for children without fast food outlets around home, adjusted for income

|  | Lower maternal education level | | | Higher maternal education level | | |
| --- | --- | --- | --- | --- | --- | --- |
|  | Persons | Person-observations | Estimate  (95% CI) | Persons | Person-observations | Estimate  (95% CI) |
| Relative fast food exposure  (+10%-point) | | | | | | |
| BMI (SDS) | 666 | 1676 | **0.04 (0.00; 0.07)** | 897 | 2268 | -0.02 (-0.04; 0.01) |
| FMI (SDS) | 514 | 1028 | 0.08 (-0.01; 0.17) | 662 | 1324 | 0.01 (-0.03; 0.04) |
| FFMI (SDS) | 514 | 1028 | 0.03 (-0.02; 0.08) | 662 | 1324 | 0.00 (-0.02; 0.02) |
| Absolute fast food exposure (+1 outlet) |  | | | | | |
| BMI (SDS) | 666 | 1676 | 0.07 (-0.01; 0.15) | 897 | 2268 | -0.06 (-0.14; 0.02) |
| FMI (SDS) | 514 | 1028 | 0.12 (-0.00; 0.24) | 662 | 1324 | -0.08 (-0.17; 0.03) |
| FFMI (SDS) | 514 | 1028 | 0.02 (-0.03; 0.06) | 662 | 1324 | -0.01 (-0.06; 0.03) |

Estimates were obtained from fixed-effects linear regression using a first-difference model specification. All analyses were adjusted for the time between measurements and net household income. Income was not collected at age 4 years, thus this analyses was restricted for the age of 6, 10, and 14 years. Missing income was imputed using income measured at the nearest time-point. BMI was collected at age 6, 10 and 14 years, FMI and FFMI at age 6 and 10 years.

Supplemental Table 3a: Associations between changes in the food environment and changes in body composition, including children that moved houses

|  | Lower maternal education level | | | Higher maternal education level | | |
| --- | --- | --- | --- | --- | --- | --- |
|  | Persons | Person-observations | Estimate  (95% CI) | Persons | Person-observations | Estimate  (95% CI) |
| Relative fast food exposure (+10%-point) |  |  |  |  |  |  |
| BMI (SDS) | 2353 | 6778 | 0.01 (-0.01; 0.02) | 2241 | 6750 | 0.00 (-0.01; 0.01) |
| FMI (SDS) | 1976 | 3952 | 0.02 (-0.01; 0.04) | 1952 | 3904 | 0.00 (-0.02; 0.02) |
| FFMI (SDS) | 1976 | 3952 | 0.01 (-0.01; 0.02) | 1952 | 3904 | 0.01 (-0.00; 0.02) |
| Absolute fast food exposure (+1 outlet) |  |  |  |  |  |  |
| BMI (SDS) | 2353 | 6778 | 0.00 (-0.04; 0.01) | 2241 | 6750 | 0.00 (-0.00; 0.01) |
| FMI (SDS) | 1976 | 3952 | 0.00 (-0.01; 0.01) | 1952 | 3904 | 0.01 (-0.01; 0.02) |
| FFMI (SDS) | 1976 | 3952 | 0.00 (-0.01; 0.01) | 1952 | 3904 | -0.00 (-0.01; 0.01) |
| Healthiness score  (+0.5 point) |  |  |  |  |  |  |
| BMI (SDS) | 2071 | 5533 | -0.00 (-0.01; 0.01) | 1883 | 5161 | 0.01 (-0.00; 0.02) |
| FMI (SDS) | 1529 | 3058 | -0.01 (-0.03; 0.01) | 1407 | 2818 | 0.00 (-0.02; 0.02) |
| FFMI (SDS) | 1529 | 3058 | -0.01 (-0.02; 0.00) | 1407 | 2818 | -0.01 (-0.02; 0.00) |

Estimates were obtained from fixed-effects linear regression using a first-difference model specification. All analyses were adjusted for the time between measurements. BMI was collected at age 4, 6, 10 and 14 years, FMI and FFMI at age 6 and 10 years.

Supplemental Table 3b: Associations between changes in the food environment and changes in body composition for children without fast food outlets around home, including children that moved houses

|  | Lower maternal education level | | | Higher maternal education level | | |
| --- | --- | --- | --- | --- | --- | --- |
|  | Persons | Person-observations | Estimate  (95% CI) | Persons | Person-observations | Estimate  (95% CI) |
| Relative fast food exposure  (+10%-point) | | | | | | |
| BMI (SDS) | 714 | 2066 | 0.02 (-0.01; 0.05) | 945 | 2796 | -0.01 (-0.03; 0.01) |
| FMI (SDS) | 628 | 1256 | 0.03 (-0.03; 0.09) | 804 | 1608 | -0.01 (-0.03; 0.01) |
| FFMI (SDS) | 628 | 1256 | **0.03 (0.00; 0.07)** | 804 | 1608 | 0.00 (-0.02; 0.02) |
| Absolute fast food exposure (+1 outlet) |  | | | | | |
| BMI (SDS) | 714 | 2066 | 0.00 (-0.02; 0.03) | 945 | 2796 | -0.01 (-0.02; 0.01) |
| FMI (SDS) | 628 | 1256 | -0.01 (-0.05; 0.02) | 804 | 1608 | -0.01 (-0.04; 0.02) |
| FFMI (SDS) | 628 | 1256 | 0.02 (-0.03; 0.06) | 804 | 1608 | -0.01 (-0.02; 0.01) |

Estimates were obtained from fixed-effects linear regression using a first-difference model specification. All analyses were adjusted for the time between measurements. BMI was collected at age 4, 6, 10 and 14 years, FMI and FFMI at age 6 and 10 years.
